# Isolated Persistent Left Superior Vena Cava Revealed by an Associated Asthma

**DOI:** 10.1155/2021/5597105

**Published:** 2021-06-19

**Authors:** I. Bachouch, N. Belloumi, M. Attia, F. Chermiti Ben Abdallah, S. Hantous Zannad, S. Fenniche

**Affiliations:** ^1^Pulmonology Department Pavilion 4, Abderrahmen Mami Hospital, Tunisia; ^2^Department of Radiology, Abderrahmen Mami Hospital, Tunisia; ^3^Faculty of Medicine of Tunis, University of Tunis El Manar, Tunisia

## Abstract

**Background:**

Persistent left superior vena cava (PLSVC) is a rare anomaly of the thoracic venous system. *Case Report*. We present a case of a patient with isolated asymptomatic PLSVC, who was diagnosed because of dyspnea revealing an associated asthma. An 18-year-old male patient complained of paroxystic sibilant dyspnea. He did not have any anomaly in physical examination. The chest X-ray revealed cardiomegaly with a widening of lower mediastinum. The electrocardiogram does not show any anomaly. Echocardiography showed the PLSVC. The thoracic contrast computed tomography of the chest showed ecstasies of the right cardiac cavities and a double superior vena cava. The patient did not have similar family cases. Respiratory functional explorations led to the diagnosis of an associated asthma. Currently, he is followed up periodically. Asthma was improved with inhaled corticosteroid treatment.

**Conclusion:**

PLSVC is rare but can have important clinical implications. Associated severe cardiac malformations must be systematically sought.

## 1. Background

Persistent left superior vena cava (PLSVC) is an uncommon vascular anomaly. It is usually asymptomatic and had no hemodynamic implications, so it is frequently detected when cardiovascular imaging is performed for an unrelated reason [1]. It affects about 0.1 to 0.5% of the general population [1]. PLSVC can be isolated or associated with other cardiac malformations, such as atrial septal defect, endocardial cushion defect, or tetralogy of Fallot [1]. In this case report, we present a patient with isolated PLSVC with no other cardiac abnormalities, who was diagnosed because of dyspnea revealing associated asthma.

## 2. Case Presentation

**Figure 1:**
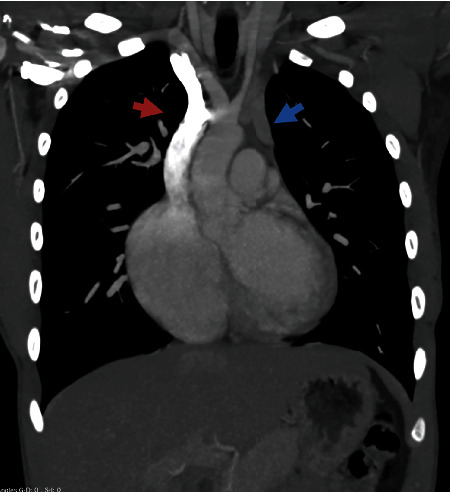
Coronal slide of the chest. Red arrow = right superior vena cava; blue arrow = persistent left superior vena cava.

**Figure 2:**
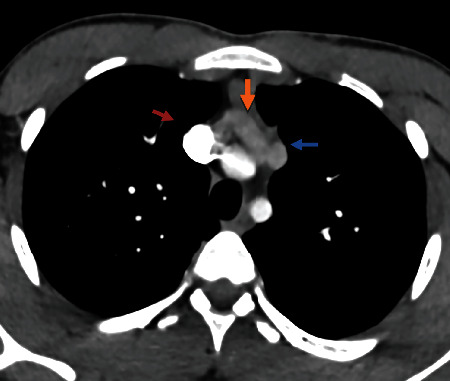
Sagittal slice of the chest. Red arrow = right superior vena cava; blue arrow = persistent left superior vena cava; orange arrow = left innominate vein.

A 18-year-old male patient presented with symptoms suggesting asthma. He was a high school student with no tobacco consumption. He did not have any exposure to bronchial irritative agents. He had a medical history of recurrent lower respiratory tract infections from birth until the age of 3 years. At the age of 13 years, paroxystic dyspnea attacks began to occur. He did not have any associated respiratory or general symptoms. Physical examination revealed normal vital signs. Respiratory, cardiac, and abdominal physical examination was normal. Complete blood count and basic metabolic panel tests were normal. The chest radiograph revealed cardiomegaly (cardiothoracic ratio = 0.6) with a widening of the lower mediastinum. The electrocardiogram does not show any anomaly. The thoracic contrast computed tomography of the chest showed ecstasies of the right cardiac cavities and a double superior vena cava (Figures 1 and 2). Echocardiography showed normal size left ventricle, dilated straight cavities without pulmonary arterial hypertension signs, no atrium septal defect, nondilated inferior vena cava, and an aspect in favor of persistent left superior vena cava. Flexible bronchoscopy showed a diffuse mucosal inflammation of the right bronchial tree without any bronchus obstruction. Endoscopy showed also the presence of a supernumerary bronchus in the right side of the right stump bronchus. The patient had normal spirometry. The methacholine challenge test showed mild bronchial hyperresponsiveness. The patient did not have similar family cases of congenital abnormalities. Currently, he is followed up periodically. By putting on 200 *μ*g inhaled beclomethasone twice daily, asthma was improved.

## 3. Discussion

PLSVC is an uncommon vascular anomaly but is considered the most common congenital anomaly of the thoracic venous system [2]. The incidence is about 0.1 to 0.5% of the total population [2].

Four anatomic types of PLSVC were described: in the first type, there is an anastomosis between the left and right superior vena cava through the innominate venous trunk. In the second type, left and right superior vena cavae are completely separated [3]. In the third type, right superior vena cava is absent or completely atrophied. In this case, blood drainage is realized through the left superior vena cava. In the fourth type, left and right superior vena cava are separated, each one presenting its own correspondent azygos vein [3]. In our case report, imaging showed the first category of the previously described classification.

Most of PLSVC are asymptomatic and have no hemodynamic abnormalities [4]. It can be detected accidentally during imaging for unrelated symptoms or in the context of a process of intravascular invasive procedure [4]. However, in some cases, there is abnormal sinus rhythm or bradycardia present in 36% of cases [5]. This cardiac rhythm disorder can lead to indication of pacemaker implantation because of sick sinus syndrome resulting from histological abnormalities caused by an enlarged coronary sinus. This arrhythmia can be caused by histological modifications in the atrioventricular sinus. The existence of multiple electric nodes between PLSVC, coronary sinus, and right atrium can also lead to this arrhythmia [6]. Tachyarrhythmia can be caused by electric generating capacity of the PLSVC, interatrial conduction delay, or atrial arrhythmia secondary to coronary sinus dilation [6]. In our case, we did not note any hemodynamic abnormalities, and electrocardiogram does not show any anomaly.

Clinical cyanosis can be also a manifestation of PLSVC (8% of patients) due to the left to right shunt. In this case, patients always suffer from septal defect, ventricular septal defect, or other cardiovascular malformations. Echocardiography in our case does not show any atrial or ventricular septal defect or other cardiovascular malformations. Contrast echography after administration of agitated saline solution should have been undertaken to document shunts between right and left cavities.

The detection of PLSVC is important when the left subclavian vein is used for catheterization procedures like Swan-Ganz catheterization and usage in renal dialysis [2, 4], in chemotherapy, or in cardiac stimulator placement [7, 8]. These investigations can be complicated by left subclavian vein thrombosis, cardiac arrhythmia, perforation of the coronary sinus, cardiac tamponade, cardiogenic shock, or even death [4].

When using a central venous catheter, drugs can directly enter in the systemic circulation when they are applied from the left brachiocephalic vein [4]. PLSVC can also be complicated by paradoxical embolism secondary to right-to-left shunt due to draining PLSVC into the right atrium either directly or via an unroofed coronary sinus [4]. A PLSVC can also cause problems in pacemaker implantation or cardiopulmonary bypass [8].

The possibility of associated severe cardiac malformations to PLSVC leads us to indicate further cardiologic investigations such as transthoracic contrast echocardiography, transesophageal echocardiography associated with contrast saline solution, MRI, or cardiac computed tomography with contrast solution [9]. The only limitation of thoracic contrast computed tomography is radiation exposure. MRI could be a best alternative significantly superior to both transthoracic and transesophageal echocardiography [9, 10]. In our case, symptoms suggest the diagnosis of asthma. However, the aspect of widening of lower mediastinum in chest radiograph (unusual in asthma patients) led us to practice thoracic contrast computed tomography that revealed the presence of PLSVC.

## 4. Conclusions

PLSVC is a very rare vascular anomaly. It is usually asymptomatic, incidentally detected after cardiovascular imaging whatever was the reason. To confirm the diagnosis, magnetic resonance imaging can be a best alternative significantly superior to both transthoracic and transesophageal echocardiography. PLSVC may have important clinical implications especially during certain catheterization procedures or in the case of associated severe cardiac malformations.

## Data Availability

Data is available on request to the corresponding author (nidhalbelloumi@gmail.com).
